# SARS-CoV-2 Variants, Current Vaccines and Therapeutic Implications for COVID-19

**DOI:** 10.3390/vaccines10091538

**Published:** 2022-09-16

**Authors:** Hong-Yu Liang, Yuyan Wu, Vicky Yau, Huan-Xin Yin, Scott Lowe, Rachel Bentley, Mubashir Ayaz Ahmed, Wenjing Zhao, Chenyu Sun

**Affiliations:** 1 The Second School of Clinical Medicine, Anhui Medical University, Hefei 230032, China; 2 The First People’s Hospital of Hefei, 390 N. Huaihe Road, Luyang District, Hefei 230061, China; 3 School of Nursing, The Philippine Women’s University, 1743 Taft Avenue, Malate, Manila 1004, Philippines; 4 Division of Oral and Maxillofacial Surgery, Columbia University Irving Medical Center, 622W 168th St, New York, NY 10032, USA; 5 College of Osteopathic Medicine, Kansas City University, 1750 Independence Ave, Kansas City, MO 64106, USA; 6 AMITA Health Saint Joseph Hospital Chicago, 2900 N. Lake Shore Drive, Chicago, IL 60657, USA; 7 Northern Jiangsu People’s Hospital Affiliated to Yangzhou University, Nantong Western Road, NO.98, Yangzhou 225000, China

**Keywords:** COVID-19, SARS-CoV-2 variants, vaccines, molecular therapeutic target

## Abstract

Over the past two years, the severe acute respiratory syndrome coronavirus 2 (SARS-CoV-2) has caused hundreds of millions of infections, resulting in an unprecedented pandemic of coronavirus disease 2019 (COVID-19). As the virus spreads through the population, ongoing mutations and adaptations are being discovered. There is now substantial clinical evidence that demonstrates the SARS-CoV-2 variants have stronger transmissibility and higher virulence compared to the wild-type strain of SARS-CoV-2. Hence, development of vaccines against SARS-CoV-2 variants to boost individual immunity has become essential. However, current treatment options are limited for COVID-19 caused by the SARS-CoV-2 variants. In this review, we describe current distribution, variation, biology, and clinical features of COVID-19 caused by SARS-CoV-2 variants (including Alpha (B.1.1.7 Lineage) variant, Beta (B.1.351 Lineage) variant, Gamma (P.1 Lineage) variant, Delta (B.1.617.2 Lineage) variant, and Omicron (B.1.1.529 Lineage) variant and others. In addition, we review currently employed vaccines in clinical or preclinical phases as well as potential targeted therapies in an attempt to provide better preventive and treatment strategies for COVID-19 caused by different SARS-CoV-2 variants.

## 1. Introduction

Coronaviruses can cause infections in a variety of animals. Two highly contagious and pathogenic members of the coronavirus family have been spread in different countries in the past two decades. In 2002, severe acute respiratory syndrome coronavirus (SARS) started in East Asia, and in 2012, Middle East respiratory syndrome coronavirus (MERS) spread in the Middle East. Then in 2019, a brand-new member of the coronavirus family, namely the acute respiratory syndrome coronavirus 2 (SARS-CoV-2), caused a disease later named as coronavirus disease 2019 (COVID-19). The COVID-19 was first reported in Wuhan, China, and it went on to fuel a global pandemic that has infected billions of people [1]. SARS-CoV-2 is a novel β-coronavirus with 79% and 50% genome sequence in homology with SARS-CoV and MERS-CoV24, respectively. At the molecular level, SARS-CoV-2 includes 6 functional open reading frames (ORFs), which are arranged in 5′-3′ order: replicase (ORF1a/ORF1b), spike protein (S), envelope protein, membrane protein (M), and nucleocapsid (N). In addition, among the structural genes, there are scattered genes encoding accessory proteins, including ORFs 3, 6, 7a, 7b, 8, and 10 [2,3]. The full-length 29,903 nucleotides of SARS-CoV-2 encode 27 viral proteins [1].

RNA viruses tend to mutate more easily due to the instability of the single-stranded RNA structure and the difficulty in correcting errors during viral replication. As COVID-19 spreads in the human population, SARS-CoV-2 has undergone multiple mutations. Virus mutation is random and may occur at any stage and site in the replication process. However, some studies have shown that mutations in the S protein have higher pathogenicity and infectivity [2,4]. The mutation of the S protein is considered to be a variant of concern (VOC) due to its greater impact on social prevalence. As a structural protein of SARS-CoV-2, the S protein mainly mediates the binding of the virus to the angiotensin-converting enzyme 2 (ACE2) receptor [5]. It is not difficult to explain why the mutation of the S protein is more likely to lead to changes in receptor affinity and virus immunogenicity.

To date, a large number of SARS-CoV-2 variants have been discovered, of which five significant variants have received extensive attention, including Alpha (B.1.1.7, Q.1-Q.8) [6], Beta (B.1.351, B.1.351.2, B.1.351.3) [7], Gamma (P.1, P.1.1, P.1.2) [8], Delta (B.1.617.2 and AY.1 sublines) and Omicron (B.1.1.529) variants [2,9]. Since the transmissibility and pathogenicity of the mutants are enhanced to varying degrees, the development of therapeutic strategies for the mutants has been widely investigated. In this review, we briefly introduce the above-mentioned five variants and summarize the current potential therapeutic strategies and molecular targets for these variants.

## 2. Predominant Variants of SARS-CoV-2 (Figure 1)

### 2.1. Alpha (B.1.1.7 Lineage) Variant

On 5 January 2021, the B.1.1.7 lineage (alpha variant) of SARS-CoV-2 was identified for the first time in the province of Los Rios by COVID-19 genomic monitors in Ecuador [10]. Through gene sequencing, it was found that the Alpha strain has 23 gene mutations in N terminal domain (NTD), RBD domain, which mainly includes D164G mutation [11], and eight mutations in the S gene (del H69/V70 (1H69/V70), del Y144 (1Y144), N501Y, A570D, P681H, T716I, S982A and D1118H) [10,12]. Among these mutations, studies have pointed out that H69/V70, N501Y and Y144 mutations can make SARS-CoV-2 more virulent and transmissible [13].

**Figure 1:**
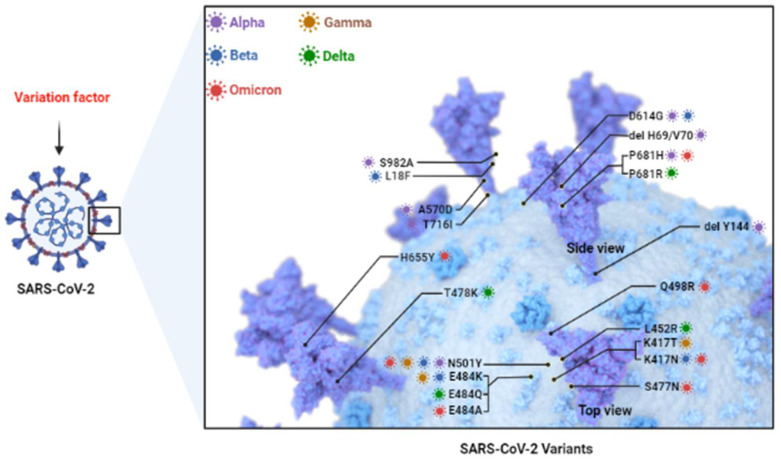
**SARS-CoV-2 Variants.** Currently common variants include Alpha, Beta, Gamma, Delta, and Omicron. They mainly consist of mutations in S protein such as D164G, N501Y, E484K, K417N, etc. (*Adapted from “The SARS-CoV-2 Variants of Concern”, by BioRender.com (2022). Retrieved from https://app.biorender.com/biorender-templates* (accessed on 28 July 2022).

H69/V70 is defined as a deletion of histidine 69 and valine 70 at the NTD site [11], which can lead to significant immune evasion after infection in immunocompromised patients and enhance the infectivity of the virus [14]. Interestingly, interactions between viral mutation sites also have the potential to impart transmissibility and virulence to the virus, as in SARS-CoV-2. Some studies have pointed out that there is a superimposed effect between H69/V70 and D614G or N439K mutations, that is, H69/V70 and D614G mutant strains exhibit faster cell–cell fusion kinetics, compared with wild-type virus strains [15]. However, due to the lack of a systemic immune barrier in the in vitro experiments, a large number of studies are needed to verify the immune escape caused by H69/V70 deletion. The N501Y mutation is defined as a tyrosine (Y) substitution of aspartic acid (N) at position 501, and its mutation site is mainly located in the receptor binding motif (RBM) region of the S gene [16,17]. As suggested above, mutations in the S region can enhance the affinity of the virus receptor to varying degrees. In the N501Y strain, the binding strength of the virus to ACE2 is significantly enhanced, and there is evidence that the affinity of the B.1.1.7 strain for ACE2 is increased by 110%, while the affinity for neutralizing antibodies is only about 70% [18]. This suggests that a booster dose is needed to cover strains with low antibody affinity, providing a new strategy for epidemic prevention policies. The current neutralizing antibodies are mainly directed against the receptor binding domain (RBD) and N terminal domain (NTD) regions of the virus S protein, but some studies have pointed out that the Y144 mutation may be involved in the failure of NTD-specific antibodies [19].

In addition to the three potential mutations mentioned above (H69/V70, N501Y and Y144), P681H is a histidine (H) mutation at the Furin cleavage site of the S protein. P681H mutation can lead to a change in the conformation of the S protein [20], which in turn leads to increased viral infectivity and affinity.

At the epidemiological level, the Alpha variant was initially prevalent in Kent, United Kingdom (due to the carrier’s history of travel), and then gradually spread in Europe. Studies have shown that the B117 variant has a reproductive number that is 43% to 90% higher than the pre-existing variant, with increased transmission in three countries, Denmark, Switzerland, and the United States, fluctuating between 59% and 74% [14]. A controlled study has also pointed out that about 70% of deaths were caused by B.1.1.7 strain infection in England [21]. In addition to the UK, studies have indicated that among the nearly 4000 COVID-19 patients in Tokat, Turkey, approximately 30% were infected with the Alpha variant. Interestingly, the Alpha variant increased the risk of hospitalization and increased mortality in the younger age group, while an opposite trend was observed in the older age group [22]. This is somewhat different from the trend in the United Kingdom, which may be due to differences in geography, the scope of popularity, and the difference in testing methods. The above evidence confirms the high transmissibility and high toxicity of the Alpha variant, but current research indicates that the currently used vaccines are still effective against the Alpha variant [23,24]. Nonetheless, the development of suitable drug targets is still imminent.

### 2.2. Beta (B.1.351 Lineage) Variant

In October 2020, SARS-CoV-2 lineage B.1.351 (also known as 501Y.V2) was first identified in the Eastern Cape province of South Africa and within a few weeks became the main endemic strain in the region [25]. This variant has multiple gene mutation sites, nine of which exist in the gene of S protein. Major mutations included K417N, E484K, N501Y and D614G, D80A, D215G, del 241, del 242, del 243, V367F, P384L, R408I, D6101G and A5101V. Among these, L18F, D80A and D215G are located in the S gene, N501Y, E484K and K417N are located in the RBD region, and A701V is located in loop 2 [13].

As previously described, mutations in the S gene tend to predispose the variant to immune evasion and increased affinity for the receptor. Therefore, E484K, N501Y and K417N play an important role in the toxicity of the Beta variants. In deep mutational scans, E484K has been shown to enhance the binding affinity of the ACE2 receptor [26]. In addition, RBD is the main target of plasma antibody neutralization activity, and studies have pointed out that the E484K mutation can reduce the efficacy of antibody therapy and lead to immune escape [27]. In addition, K417N has also been shown to induce a conformational change of the S protein, making it difficult for the virus to be recognized by antibodies [28], and also increasing the infectivity of the virus [29]. The study noted that K417N was more than 100-fold less sensitive to etesevimab129 and about 10-fold less sensitive to casirivim, but remained unchanged to bamlanivimab, imdemab, and sotrovimab [30,31]. Neutralization of Beta variants by antibodies is diminished due to mutations in multiple S genes, leading to concerns about the effectiveness of the vaccine. In fact, Cele et al., have suggested that B.1.351 was able to escape an antibody response to infection initiated by the early variant at an early stage [32]. In addition, the E484K mutation in the Beta variant includes a nucleotide substitution of G23012A, which is thought to be involved in altering the antigenicity of the virus, resulting in a less effective vaccine [24,27,33]. Wang et al., have also demonstrated that B.1.351 is resistant to neutralization by mAbs directed against NTD super locations, and is also resistant to the main group of effective mAbs directed against RBM [31]. All of the above findings suggest that the viral transmissibility and current neutralizing antibodies that result from S gene mutations are less potent. However, the vaccines currently in existence remain effective against most variants including Beta variant, which reduces public fear.

At the epidemiological level, the B.1.351 lineage was first identified in Cape Town, South Africa and led to a second wave of epidemics. In addition to South Africa, 96% of the 23 individual samples collected in Zambia during mid-December 2020 were the B.1.351 variant. Of the 245 genomes previously sequenced, none came from this lineage [7]. In addition to Africa, B.1.351 variant cases have also been reported in many European countries (Belgium and Austria, etc.), and about 350 cases have been found. Some of these cases have a history of international travel, but infections without epidemiological links are also increasing. In addition, according to the Centers for Disease Control and Prevention (CDC), cases of the B.1.351 variant had emerged in 36 U.S. states by 22 March 2021. These data underscore the strength of the B.1.351 lineage spread. However, there is still a lack of information on the influence of the B.1.351 lineage on the severity of COVID-19 pneumonia.

### 2.3. Gamma (P.1 Lineage) Variant

The gamma variant P.1 lineage was first detected in four passengers travelling from Brazil to Tokyo during a routine screening at Tokyo Airport in Japan in January 2021 [34]. Gamma variant contains RBD mutations N501Y, E484K and K417T, in addition to nine mutations in the S protein, L18F, T20N, P26S, D138Y, R190S, D614G, H655Y, T1027I and V1176F substitutions [35,36]. Among these, N501Y and E484K have appeared in Alpha (N501Y) and Beta (N501Y, E484K), while K417T is similar to K417N in Beta. In fact, K417T differs from other mutations in that it rarely occurs in the absence of other receptor-binding motif (RBM) mutations, probably because the K417 mutation reduces ACE2 binding [23,35]. In addition to N501Y, E484K and K417T, L18F at the NTD has been shown to interfere with the binding of NTD-targeted neutralizing antibodies [37]. This adds to its action against neutralizing antibodies, and the Gamma variant has been reported to have similar resistance to FDA-approved monoclonal antibodies (mAbs) as the Beta variant [38,39,40,41]. However, interestingly, the data of Dejnirattisai et al., show that P.1 is significantly less resistant to naturally acquired or vaccine-induced antibody responses than B.1.351 [38], which also demonstrated the role of mutations other than RBD on neutralizing antibodies. In addition to its effects on neutralizing antibodies, P.1 also has a certain impact on the severity of COVID-19. The study noted that it caused a 3–4-times higher level of the viral load than previous variants and resulted in an estimated 1.1- to 1.8-times higher mortality rate [42]. In addition, in Manaus, Brazil, the Gamma variant spread about twice as fast as the previous variant, and it increased the risk of reinfection compared to the previous variant [42]. At the epidemiological level, patients infected with the P.1 variant had a higher transmission intensity due to higher viral loads in Gamma variant and mutations in the RBD. Of the confirmed coronavirus infections in Umbria, Italy, more than 51.1% of the cases had the SARS-CoV-2 P.1 variant, according to the study [35].

### 2.4. Delta (B.1.617.2 Lineage) Variant

B.1.617.2 is considered to be the most concerning variant at present, which shares a common ancestor with Kappa (B.1.617.1). Delta was first discovered in India and then named Delta variant by the World Health Organization (WHO). The Delta mutation caused the second deadly wave of COVID-19 infections in India in April 2021 [43]. Before that, the variant was first detected in the United States in March 2021; then the Delta variant rose to become a common strain in the following weeks. Delta variants have been reported to contain mainly RBD mutations L452R and T478K, a mutation P681R proximal to the FURIN cleavage site, and several mutations within orf3, orf7a, and nucleocapsid genes [44,45]. Of these, the T478K and L452R were considered the most meaningful variant, which largely contributed to the Delta pandemic. T478K was a common variant in the Mexican region by January 2021 [46,47]. Unlike most other RBD mutations, T478K retains sensitivity to all but a few mAbs, and to most convalescent and post-vaccination plasma samples [27,30,48,49]. In addition to T478K and L452R, the P681R mutation also contributed to the increased transmissibility of the virus. P681R occurs at the cleavage site of the S1–S2 subunits and facilitates the interaction with Furin, which drives membrane fusion and facilitates viral transmissibility [50]. Furthermore, it has been demonstrated in vitro that deletion of the Furin protease cleavage site in SARS-CoV-2 amplifies replication in Vero cells, but attenuates replication in respiratory cells and pathogenesis in vivo [51]. This suggests a role for P681R in viral load and viral pathogenicity and leads to longer virus shedding in throat swab samples.

It is worth exploring whether patients infected with the Delta variant remain sensitive to convalescent and post-vaccination plasma with these mutations. In fact, Planas et al., isolated an infectious strain of the Delta variant from a COVID-19 patient who had returned to France from India, with subsequent data showing that the Delta variant was resistant to several anti-NTD and anti-RBD mAbs (including bamlanivimab), and these antibodies showed impaired binding to the spike protein. In addition, sera from individuals who received either the Pfizer or AstraZeneca vaccine had significantly decreased inhibitory effect on the Delta variant [52]. Further data showed that the Delta variant resulted in approximately a 3- to 10-fold reduction in sensitivity to 45% of convalescent plasma samples and a >10-fold reduction in sensitivity to 5% of convalescent plasma samples [53,54]. In addition, there is some evidence that Delta variant may lead to more severe disease than the ancestral virus in unvaccinated subjects: the infection rate of the Delta variant is significantly increased in the unvaccinated population [55]. This situation was stressful for many developing countries, especially countries with low vaccination rates, which allowed the Delta variant to spread faster and cause more mutations. In addition, some studies have shown that the efficacy of vaccination in patients with the Delta variant infection decreases over time. The efficacy of the BNT162b2 vaccine at 120 and 150 days of vaccination was reported to be 85% and 73% respectively [56].

Now the characteristics of the Delta variant can be roughly understood: it has greater transmissibility, higher viral load, and a shorter incubation period. In terms of transmissibility, the R0 of the Delta variant fluctuates between 5 and 9.5, and it spreads faster than MERS and SARS, smallpox, the common cold, Spanish flu, and Ebola [57]. Much of this high infection rate is due to Delta’s higher viral load: Delta’s viral load has been reported to be about 1000 times that of the original strain [58]. In addition, studies have pointed out that, compared to the wildtype (WT) strain, the virus clearance rate of the Delta variant is slower and the risk of deterioration to critical state is higher than that of the WT infection [59]. In Canada, the Delta variant has been associated with higher hospitalization and mortality; in Singapore, the Delta variant has been associated with higher oxygen demand and increased incidence of pneumonia; in Scotland, the Delta variant has also been associated with higher hospitalizations rate [60].

It is worth noting that with the widespread spread of the Delta variant, a variant of the Delta plus (also known as AY.1 or B.1.617.2.1) has since appeared. It was first discovered in India and gradually spread [61]. Furthermore, compared with the original Delta (B.1.617.2), the Delta plus variant has six key mutations, T95I, G142D, R158G, L452R, T478K and K417N. Notably, it has been reported that the K417N mutation appears to lead to immune evasion by losing the interaction of K417 with Y52, thereby reducing antibody binding to the S protein [61]. This has obviously brought a lot of pressure to the prevention and control of COVID-19 in some countries highly impacted by Delta variant.

### 2.5. Omicron (B.1.1.529 Lineage) Variant

On 24 November 2021, a new B.1.1.529 variant emerged and was first identified from an international traveler in South Africa [62]. It was designated as a variant of concern (VOC) by the WHO on 26 November 2021. Surprisingly, it was later discovered that the Netherlands had found its first Omicron-positive patient a week before the announcement of Omicron infection, adding to the difficulty of identifying the first patient [63]. The Omicron variant of the SARS-CoV-2 genome consists of 18,261 mutation sites, of which more than 97% are located in the coding region, and the remaining 558 mutations are located in the extragenic region [64]. Among these, 37 mutations have been found to be located in the RBD region which are more meaningful for virus evasion of antibody neutralization and immune evasion, and they mainly include K417N, S477N, Q498R, E484A and N501Y. There are also some mutations at the Furin site, namely H655Y, N679K and P681H, which provide more power for virus replication and entry into cells [2]. Besides in RBD, there are 11 mutations in the NTD region, including six deletions and one insertion, of which the N211 and ins214EPE mutations are unique to the Omicron [65]. Among these, ins214EPE has previously appeared in seasonal coronaviruses such as HcoV-229E.

On an epidemiological level, Omicron’s contagion rate is appalling. As the omicron spread around the world within just days to weeks, so did the attention. According to the CDC report, during December 2021, the Omicron variant increased by 2.5% within two weeks in the United States, and the infection rate was about 13% in the New York/New Jersey area [63]. In the UK, cases of the Omicron variant doubled every two to three days [63]. Behind such high infection rates is the number and variety of mutations. Multiple mutations located in the RBD increase binding affinity to the ACE2 receptor, which is known to be a major contributor to increased transmission rates. In addition to having a very high infection rate, studies have shown that Omicron increases the risk of reinfection [66]. In order to reduce the prevalence of Omicron in various regions of the world, it is recommended that vaccination be accelerated as soon as possible in vulnerable regions of the world.

Since the emergence of Omicron in patients who have been vaccinated against COVID-19 [67], concerns have arisen about the efficacy of Omicron’s vaccine and higher demands have been placed on the therapeutic antibodies that currently exist. A recent study has noted that Omicron, as expected, is resistant to most therapeutic antibodies, but remains susceptible to inhibition by sotrovimab, and that double immunization with BNT162b2 may not adequately prevent severe disease induced by this variant [65]. Only 20% and 24% of detectable neutralizing antibodies against Omicron variants HKU691 and HKU344-R346K, respectively, were present in BNT162b2 recipients [68]. In addition to BNT162b2, laboratory studies with the Pfizer-BioNTech vaccine have shown a high level of protection with three doses of the Omicron variant [63], but the results still warrant further exploration. Due to the large-scale decline in the protective effect of vaccines, it is necessary to develop new vaccines or molecular targeted therapies in the future, which poses greater challenges for the treatment of COVID-19.

### 2.6. Other Variants

#### 2.6.1. Lambda Variant

In April 2021, a patient infected with a new variant of Lambda (c.37) was first reported in Lima, the capital of Peru [9]. The Lambda variant was subsequently defined by WHO as a variant of interest (VOI). Lambda has become the predominant strain in Peru and has subsequently spread to other countries. Genomic data indicate that Lambda has several major mutations, including G75V, T76I, del 246-252, L452Q, F490S, D614G, and T859N. Among these, L452Q and F490S are unique to Lambda. The study noted that the L452Q mutation enhanced Lambda’s affinity for ACE2 and doubled the infectivity of the strain [69].

#### 2.6.2. Mu Variant

In addition to Lambda, the Mu variant has also been identified as a type of VOI. The Mu variant was first discovered in Colombia in January 2021 and has been scattered in various regions since then. Mu variant mainly includes INS 146N, Y144T, Y145S, R346K, E484K, N501Y and P681H. Among these, like the other variants, E484K and N501Y confer greater infectivity and antibody evasion to Mu. However, studies have shown that neutralization of serum antibodies to Mu variant in individuals vaccinated with two doses of BNT162b2 remains robust and effective, although at lower levels than other B.1 lineages of SARS-CoV-2 [70]. As of November 2021, the Mu variant virus has spread to nearly eight countries, including Colombia, the United States, Spain, the Netherlands, Denmark, Mexico, Germany and Curacao [2]. Although there is a small amount of research showing that the current vaccine is still effective against the Mu variant, it is still necessary to remain vigilant. Although, when compared to VOCs, the VOIs are less likely to cause widespread and high mortality and hospitalization rates, more in-depth exploration of VOIs is required in the future due to their potential to further mutate into VOCs.

#### 2.6.3. Kappa Variant

Recently, the WHO proposed the concept of variants under surveillance (VUM), which refers to variants with several heritable changes that are speculated to affect the characteristics of the virus and may pose a threat to the public in the future. These mainly include variants such as Kappa, Epsilon, Iota, Eta and etc. The Kappa variant (B.1.617.1), first discovered in India in December 2021 [71], originated from the same ancestor as the Delta variant. The genomic data indicate that the mutations on the S protein of the Kappa variant mainly included L452R, T478K, E484Q, D614G and P681R. Compared to Delta, the Kappa variant possesses higher host infectivity and leads to stronger immune escape [50]. Studies indicate that both L452R and E484Q mutations make Kappa variants resistant to antibody neutralization [72,73,74]. Luckily, the data of Yang et al., show that the RBD vaccine is still effective against Kappa variant in mice, although its antibody neutralizing titer is 3.5-fold lower than that of the wild-type strain [75]. In addition to the RBD vaccine, injection of BNT162b2-induced neutralizing serum or injection of mRNA-1273-induced neutralizing serum also remains effective against Kappa variants [53,71]. These findings reduce the doubts about the effectiveness of current vaccines (Table 1).

## 3. Current Vaccine

Currently, vaccines against COVID-19 have been extensively developed, including Pfizer/BioNTech mRNA vaccine, Moderna mRNA vaccine, Oxford-AstraZeneca (AZD1222) vaccine, CoronaVac vaccine, DNA vaccine, Beijing Institute of Biological Products inactivated vaccine (BBIBP-COV), Zhifei Longcom recombinant protein vaccine (ZF2001), protein subunit vaccine, etc. [76]. Here we provide a brief introduction to the vaccines that currently exist.

### 3.1. Pfizer/BioNTech mRNA Vaccine

The BNT162b2 vaccine induces the body to produce neutralizing antibodies by transcribing and translating the S protein to induce protection. According to the data, BNT162b2 has 95% protection against COVID-19, and the adverse reaction rate is extremely low [77]. This affirms the efficacy of the BNT162b2 vaccine in patients naturally infected with the wild-type strain. However, since the mRNA sequence of BNT162b2 was developed on the basis of the original WT virus strain, it is worth exploring whether it has a preventive effect on the currently prevalent SARS-CoV-2 variant. The data show that the neutralization titers of BNT162b2 vaccine-primed human sera against major S protein mutations such as 69/70 deletion, E484K and N501Y are 0.81- to 1.46-times higher than the original strain, with little effect of the mutation [78]. In addition, a second dose of BNT162b2 vaccine has also been shown to neutralize B.1.617.1 (Kappa), B.1.617.2 (Delta) variants two-to-four weeks after vaccination [53]. In addition, BNT162b2 has also been shown to have a neutralizing effect on VOCs such as Alpha, Beta, and Gamma [78,79,80]. In the real world, a clinical study in Scotland noted that the BNT162b2 vaccine reduced COVID-19 hospitalizations 28–34 days after vaccination [81]. Additionally, among the nearly one million members who received two doses of BNT162b2 between January and February 2021, the vaccine following the third dose provided additional protection against SARS-CoV-2 infection in those vaccinated six months earlier [82]. This provides new recommendations for scientific vaccination. The above data all show that the BNT162b2 vaccine is currently effective, but there is another issue of concern which is the safety of the vaccine. In the clinical trial of Polack et al., the BNT162b2 vaccine was found to have a low incidence of adverse reactions, mainly including short-term, mild to moderate pain at the injection site, fatigue, and headache [77]. Results from a cohort study in the United States indicated a low probability of adverse event risk within 42 days of the first dose of BNT162b2. There was little difference in the risk of adverse events within 14 days after the first dose of BNT162b2 or mRNA-1273 vaccine [83]. However, in an Israeli study, it was found that nearly 1–5 per 100,000 people in BNT162b2 vaccine recipients developed myocarditis, and there were other serious adverse reactions including pericarditis, arrhythmia, deep vein thrombosis, pulmonary embolism, etc. [84]. This suggests that although the incidence is low, serious adverse reactions still occur.

### 3.2. Moderna mRNA-1273 Vaccine

Moderna mRNA-1273 is an mRNA vaccine which uses the segments of SARS-CoV-2 hereditary material to induce the body to translate and express stabilized spike protein and produce corresponding antibodies for neutralization. The most direct indicator for evaluating a vaccine is its degree of disease prevention. For the Moderna vaccine, studies have shown that it has 94.1% efficacy against COVID-19 [85]. This is similar to the efficacy of the Pfizer/BioNTech vaccine. For different SARS-CoV-2 variants, Chemaitelly et al., have shown that the Moderna mRNA-1273 vaccine is very efficient (96.4%) against the Beta variant and even exceeds its efficacy against the wild strain. Additional studies have also found that Moderna mRNA-1273 vaccine efficacy was negligible for two weeks after the first dose but increased rapidly in the third and fourth weeks before the second dose [86]. In addition to its high neutralization efficiency against Beta, it is 89% effective against Alpha variants and 85% effective against Gamma variants [87]. However, it is worth further exploring the way in which some studies have pointed out that the Moderna mRNA-1273 vaccine is 85% effective against the Beta variant [87]. In any case, the Moderna mRNA-1273 vaccine has high efficacy for Alpha, Beta, and Gamma, which reduces the pressure of updating the vaccine. Moreover, for the highly infectious Delta variant, the Moderna mRNA-1273 vaccine was only 50.6% effective [88]. It is noteworthy that the use of mRNA-1273 following a primary course of ChAdOx1 nCoV-19 or BNT162b2 has been shown to significantly increase protective effect against Omicron variant [89]. It is also encouraging that the three doses of vaccine provide better protection for Omicron than the two doses [90]. In terms of vaccine safety, the probability of adverse reactions of the Moderna mRNA-1273 vaccine is generally low [91]. Phase 3 trials have shown that the probability of all adverse reactions after the first dose is 2.9% and 15.8% after the second dose. Minor hypersensitivity reactions such as allergies and pain at the injection site are the main symptoms [85].

### 3.3. AZD1222 Vaccine

AZD1222 is a ChAdOx1 vaccine using chimpanzee adenovirus as a carrier. After entering the body, it expresses a natural-like S protein and induces the body to produce neutralizing antibodies [92]. For the original SARS-CoV-2 infection, the effective rate of AZD1222 was 62.1–79%, which may be due to its unstable transcriptional expression in humans [93,94]. For the Beta variant, a 2020 clinical trial showed that AZD1222 was only 10.4% effective against the Beta variant and did not show efficacy against Beta variant-infected COVID-19 after two doses [95]. This suggests that countries or populations vaccinated with AZD1222 need further vaccinations or other therapeutic strategies to prevent Beta variants. The good news is that two doses of AZD1222 were 74.5% effective against the Alpha variant and 67% effective against the Delta variant [96]. In addition, the safety issues of the AZD1222 vaccine cannot be ignored. There are many reports that the hematological adverse reactions occurred after vaccination with AZD1222, including thrombotic thrombocytopenia [97,98,99], thromboembolism [100], thrombosis [101], etc., as well as skin diseases such as rash and psoriasis [102,103,104]. This serious adverse reaction and low neutralization efficiency forced people to search for a safer and more effective AZD1222 vaccination regimen. The study indicated that ChAdOx1 nCoV-19/BNT162b2 mRNA heterologous vaccination could enhance the neutralization efficiency to 91.6%, which was significantly higher than that of BNT/BNT homologous vaccination [105].

### 3.4. CoronaVac Vaccine

The CoronaVac vaccine is an inactivated vaccine and its immunogen is the SARS-CoV-2 strain. By using certain means, the virus is inactivated, but a certain immunogenicity is retained, and the body can be induced to produce corresponding neutralizing antibodies against the inactivated S protein in vivo. Compared with nucleic acid vaccines, CoronaVac vaccines have the advantages of large yield, safety, and simple transportation and storage. However, due to the slow production of antibodies, multiple doses of vaccination are often required. A phase 3 trial in Turkey showed that the CoronaVac vaccine is 83.5% effective in patients aged 18–59 [106]. For different SARS-CoV-2 variants, the data show positive rates of neutralizing antibodies in the population immunized with CoronaVac to be over 80% for the Alpha and Gamma variants, over 75% for the Delta variant, and over 60% for the Beta variant [107,108]. In a clinical study conducted in Brazil, vaccination with CoronaVac was associated with a reduction in COVID-19 symptoms, hospitalizations and deaths in adults over 70 years of age in the presence of widespread gamma variants. The anti-hospitalization rate was 77.6%, and the anti-mortality rate was 83.9% [109]. This suggests that the CoronaVac vaccine has varying degrees of protection against these different variants. There are data showing that Omicron escape neutralizing antibodies elicited by the CoronaVac vaccine, suggesting that CoronaVac is insufficiently effective against Omicron [68]. However, Perez-Then et al., pointed out that the heterologous vaccination to combine BNT162b2 and Coronavac can improve the protection efficiency of Omicron [110]. In addition, it is worth noting that Coronavac is testing an Omicron-specific strain of the vaccine, which has been approved for human trials in Hong Kong [68]. In addition, in a phase 1/2 clinical trial, researchers in China suggested that the CoronaVac vaccine was indeed safe and well tolerated, could induce humoral responses, and produced higher neutralizing antibody titers at a dose of 3.0 micrograms [111].

### 3.5. DNA Vaccine

DNA vaccine is a piece of plasmid DNA encoding an immunogen, which can directly induce transcription and translation in the body and induce corresponding neutralizing antibodies. On the one hand, DNA is more convenient to store and does not need to be frozen, and on the other hand, it will not induce the body to produce an anti-carrier response. As a DNA vaccine targeting the full-length S protein of SARS-CoV-2, INO-4800 was shown in Phase 1 clinical trials to stably induce humoral and cellular immunity and secrete antibodies, showing excellent efficacy and safety [112]. In silico biomolecular modeling results show that the vaccine-induced neutralizing antibodies produced by INO-4800 were still effective against G614 and Alpha but were less potent against the Beta variant [113]. In addition, in addition to the INO-4800 vaccine, ZyCoV-D is also considered to be a highly efficient DNA vaccine. Potential immunogenic potential has been shown in earlier animal models [114]. Results of a multicenter, double-blind, randomized, placebo-controlled Phase 3 trial in 49 centers in India showed a vaccine efficacy of 66.6%. And in terms of safety, the results of the Phase 3 clinical trial showed that the incidence of adverse events in the treatment group was similar to that in the placebo group [115].

### 3.6. NVX CoV-2373 Vaccine

The NVX CoV-2373 vaccine refers to recombinant nanoparticles targeting the SARS-CoV-2 S protein, and its immunogen is the adult SARS-CoV-2 S protein particle. After entering the body, it can induce the body to produce an antiviral neutralization response. Results from a phase 3 clinical trial in the UK showed that a two-dose NVX-CoV-2373 vaccine regimen administered to adult participants was 89.7% protective against SARS-CoV-2 infection [116]. A subsequent study also indicated that the NVX-CoV-2373 vaccine was 90% protective against COVID-19 seven days after the second dose [117]. The study noted that higher vaccine efficacy was observed with the NVX-CoV-2373 vaccine in HIV-negative participants and 92.7% of infections were caused by the Beta variant [118]. The NVX-CoV-2373 vaccine is currently one of the main vaccines against the Beta variant due to the high rate of protection against the Beta variant shown by the NVX-CoV-2373 vaccine. In addition, the study by Paul T Heath et al., also pointed out that the efficacy of the NVX-CoV-2373 vaccine was 86.3% for the Alpha variant and 96.4% for the non-B.1.1 variant [116]. The results from the US–Mexico study showed 92.6% efficacy of the NVX-CoV-2373 vaccine against the variant of interest or interest [119]. In terms of vaccine safety, the findings show that reactogenicity of NVX-CoV-2373 vaccines is generally mild and transient. The incidence of serious adverse events was lower and similar in the placebo and vaccine groups [116]. Further, in young and old people of different ages, the two-dose 5 μg vaccination regimen is safe and effective [120]. The high safety profile and efficacy provide the basis for the NVX vaccine to be administered in areas where the Alpha and Beta variants are prevalent (Table 2).

## 4. Potential Molecular Therapeutic Target (Table 3)

### 4.1. S Protein

The S protein is located on the surface of the virus and is mainly responsible for the entry of the virus into the host cell. It binds primarily to the human ACE2 receptor, which is often the first step in a SARS-CoV-2 attack on human cells. At the molecular level, the S protein is composed of two subunits, namely S1 and S2, and each S protein is composed of 3 S1/S2 subunits, forming a trimer. The RBD of the S1 subunit is responsible for binding to ACE2 [123]. RBD switches between a “closed” or “down” receptor-inaccessible conformation, and an “open” or “up” conformation that allows binding to the ACE2 receptor [124,125]. Thus, mutations in the S protein may facilitate the entry of the virus into the host, leading to increased infectivity and affinity of the virus, which is the main form of the current mutation. Some studies have found that the D614G mutation in the spine region at position 23,403 of the WT strain caused by a single base change from G to A is the main pandemic form of the current pandemic [126]. With a reasonable blockade of the S protein, the virus can be prevented from binding to the host. Therefore, targeting the S protein is considered to be the most promising pharmacological treatment at present. The main method currently adopted is to block the S protein by injecting mAbs or by activating artificiality acquired active immunity to induce neutralizing antibodies by injecting vaccines. This in turn inhibits the binding of the virus to the host cell.

**Table 3:** Potential molecular therapeutic target.

Pharmacological Target	Role of Target in Viral Infection	Therapeutic Strategy	Drug Candidate
S protein	Binds to ACE2, mediates virus-host fusion	block	Monoclonal antibodies (such as bamlanimab, etesevimab, etc.) or vaccines
ACE2	Binds to S protein, mediates virus-host fusion; Mediates Ang-II-induced toxicity during COVID-19	block	infusion of transgenic human recombinant soluble ACE2 (hrsACE2) or circulating extracellular vesicle ACE2 (evACE2)
IL-6	Causes tissue damage and promotes a flare-up of inflammation	inhibit	tocilizumab
IL-1		inhibit	anakinra
TNF-α		inhibit	Adalimumab; N-acetylcysteine; tramadol
M^Pro^	Facilitates the processing of viral proteins	inhibit	nirmatrelvir
TMPRSS2	Responsible for cleaving the S protein and promoting the binding of ACE2 to the S protein	inhibit	Camostat; nafamostat

The RBD region of the S protein is the primary target of neutralizing antibodies. To show the efficacy of neutralizing antibodies, the results of early animal studies showed adoptive transfer of purified IgG in convalescent rhesus monkeys protected naive recipient macaques from SARS-CoV-2 challenge in a dose-dependent manner [127]. Furthermore, there are studies that suggest that neutralizing antibodies may reduce the severity of infection as well [128]. One study noted a significant reduction in SARS-CoV-2 viral load on day 11 following treatment with bamlanivimab and etesevimab [128]. Anti-RBD immune IgG has also been considered a predictor of disease severity and survival [129]. The above research results all reveal the efficacy of antibodies against the RBD region of the S protein. In fact, in addition to RBD, there are antibodies against the NTD region. It inhibits viral binding to cells mainly by binding to the largest sugar-free surface of the NTD away from the viral membrane (called the NTD supertope), resulting in inactivation of the S protein [130]. It has been reported that neutralizing antibodies against NTD targets have strong binding to SARS-CoV-2 and can protect Syrian hamsters from SARS-CoV-2 challenge [37]. Except for RBD and NTD, there are some neutralizing antibodies that bind to other regions of the S protein, including the C-terminal domain and the S2 [131,132], but the effects of these antibodies are still worth exploring. Using an overlapping library of linear B-cell peptides, earlier researchers have reported two IgG immunodominant regions on the SARS-CoV-2 spike glycoprotein, which are located near the RBD. These regions were identified by sera from patients recovering from COVID-19. Antibody depletion assays showed that antibodies targeting these immunodominant regions significantly altered virus neutralization capacity [133]. The above studies have demonstrated the efficacy of RBD, NTD and other region-targeted neutralizing antibodies in COVID-19. Although there has been some progress in the development of antibodies, in view of the prevalence of S protein mutations, it is still necessary to find more conserved neutralizing epitopes of SARS-CoV-2. In addition, due to the complexity of the human body, the development of antibodies requires more clinical studies for verification. Additionally, studies have shown that cellular immune responses may help protect against SARS-CoV-2 if the antibody response is suboptimal [127,134].

### 4.2. ACE2

The renin-angiotensin-aldosterone system (RAAS) is an important polypeptide network that regulates physiological states such as blood pressure, osmotic pressure and urine output in the human body. It is composed of a variety of signaling proteins and receptors. The ACE2 receptor is at the heart of the RAAS system. Human ACE2 is an 805 amino acid polypeptide, which is essentially a membrane carboxypeptidase that cleaves a hydrophobic residue from the C-terminus of the substrate. ACE2 binds to the cell membrane and functions through a hydrophobic region close to the C-terminus of the substrate, and its active site is mainly located in its own N-terminal region, facing the extracellular space [135,136,137]. ACE2 degrades angiotensin II (Ang II) to angiotensin, which promotes vasoconstriction and sodium retention. Furthermore, ACE2 plays an important role in a range of inflammatory responses [138]. It is well known that one of the most important symptoms after the onset of COVID-19 are respiratory tract infections, including the release of inflammatory mediators in the lungs and damage to the pulmonary blood vessels and interstitium. Previous studies have suggested that ACE2 might be able to attenuate lung damage and vascular damage [139,140] and further reduce pulmonary fibrosis and improve cardiac function [141,142]. So, would the symptoms of COVID-19 be improved by injecting ACE2? In fact, there are also clinical research data confirming that the use of recombinant human ACE2 (rhACE2) can significantly reduce plasma inflammatory markers 2–4 h after treatment [143]. The above studies suggest the protective role of ACE2 in lung infection and injury, and during COVID-19, it is recognized that the cell surface amount of ACE2 decreases in patients [144]. Therefore, appropriate infusion of ACE2 may be a therapeutic approach targeting ACE2. There are two main mechanisms by which rhACE2 treats COVID-19. One is to compete with the S protein of SARS-CoV-2 to inhibit the combination of the virus and cells; the other is to regulate the RAAS system to relieve inflammation and tissue damage caused by Ang-II and other substances [145,146].

ACE2 as an important factor in the inflammatory response and regulation of osmotic pressure in vivo, there are two main forms of ACE2 in human body, namely membrane-bound ACE2 and circulating ACE2. SARS-CoV2 is mainly bound to the N-terminus of membrane-type ACE2 [147]. The currently developed treatment options for COVID-19 mainly include the infusion of transgenic human recombinant soluble ACE2 (hrsACE2). Soluble ACE2 has the ability to bind to SARS-CoV-2 because it contains a viral binding site, which thereby competes the virus from binding to cellular ACE2 [144]. In fact, numerous studies have shown that hrsACE2 can reduce viral titers in infected patients and can significantly shorten recovery time [148]. Clinical study data show that hrsACE2 reduces SARS-CoV-2 recovery in Vero cells by 1000–5000-fold, and hrsACE2 can significantly block the early stages of SARS-CoV-2 infection [149]. This provides new treatment recommendations for patients who have just been infected with COVID-19. A case report of the use of hrsACE2 nine days after the hospitalization of a critically ill patient showed that the clinical symptoms of the patient improved significantly in the following week, and the patient finally recovered and was discharged from the hospital. This suggests the feasibility of using hrsACE2 in critically ill patients [148,150]. Recent studies have also proposed that the combination of hrsACE2 and remdesivir can treat COVID-19 with multiple mechanisms. The two drugs show a strong additive effect at sub-toxic concentrations, inhibiting both the binding of virus to cells and the replication of viral RNA [151]. In addition to the use of hrsACE2, a new mode of ACE2 infusion has recently been proposed—circulating extracellular vesicle ACE2 (evACE2) [152]. Studies have shown that compared with recombinant human ACE2 without vesicles, evACE2 is effective in blocking the viral spike protein. RBD exhibited a 135-fold higher potency in binding and protected transgenic mice from SARS-CoV-2-induced lung injury and death. Most surprisingly, the researchers also evaluated the efficacy of evACE2 in inhibiting infection with SARS-CoV-2 variants (Alpha, Beta and Delta) equal to or higher than the wild-type strain [152]. The high protective effect of evACE2 may be due to the existence of vesicles that prevent ACE2 from being attacked or degraded by autologous substances in the circulation, which in turn can exert better biological effects. Recent studies have proposed that the combination of hrsACE2 and remdesivir can treat COVID-19 with multiple mechanisms. The two drugs show a strong additive effect at sub-toxic concentrations, inhibiting both the binding of virus to cells and the replication of viral RNA [151].

In addition to direct infusion of ACE2, microRNA (miRNA) therapy targeting the ACE2 gene has also been developed recently. The miRNAs are endogenous non-coding small RNAs composed of nearly 22 nucleotides that can regulate one-third of human gene expression. It is known that miRNAs can affect a variety of cellular signal transduction pathways, thereby participating in various pathophysiological conditions [153]. Currently, the miRNAs targeting ACE2 under investigation include miRNA429, hsa-miRNA200c-3p, and hsa-miRNA200b-3p [154]. Members of the miR-200 family, especially miR-200c-3p, were found to be strong candidate targets for regulation of ACE2 by further target prediction algorithms [155]. Although the treatment of miRNA targeting ACE2 has been put on the agenda, additional cell lines and animal models are still needed in the future to study the efficacy and adverse reactions of miRNA therapy during the treatment of COVID-19. In any case, ACE2 is an important target for virus invasion and self-protection, and it is also the most potential drug development target at present.

### 4.3. Inflammatory Cytokine

Cytokines are mostly signaling molecules composed of various proteins and glycoproteins secreted by many cells to transmit signals between cells. Cytokines play a huge role in the host’s infection, trauma, and inflammatory responses. Cytokine storm is a cascade of immune inflammatory response that eventually leads to the massive release of cytokines [156]. After infection, the SARS-CoV-2 replicates rapidly in the host cells, producing a large number of progeny viruses, and causing the host cells to release a large number of inflammatory mediators. In addition, SARS-CoV-2 can also stimulate T cells or B cells with viral antigens, and release a large number of neutralizing antibodies and cytokines, resulting in a sudden increase in the concentration of cytokines in the body, forming a cytokine storm [157]. These cytokines will cause the immune system to produce an excessive attack on the human body and cause severe pneumonia, which mainly include interleukin-6 (IL-6), interleukin-1 (IL-1), tumor necrosis factor-α (TNF-α), and so on [158]. These cytokines play biological roles by binding to receptors on the surface of tissue cells> Therefore, the question as to whether blocking the cytokine storm during COVID-19 would have certain effects merit further investigation. In addition, there is substantial evidence that variant-induced COVID-19 may lead to a more severe inflammatory cytokine storm [159,160], so targeting cytokines may be possible regardless of the strain of COVID-19 that causes the most infections as a potential therapeutic strategy.

#### 4.3.1. Anti IL-6

As a pro-inflammatory factor, IL-6 is involved in immune response, inflammation, hematopoiesis and embryonic development, etc. IL-6 can mediate the development of pneumonia and lead to further inflammation [161]. The rise in IL-6 during COVID-19 has been confirmed by numerous studies [162,163]. Current therapeutic strategies reduce the biological effects of IL-6 by blocking the IL-6 receptor (IL-6R) with tocilizumab. However, a meta-analysis published in 2021 showed that the current treatment of tocilizumab in IL-6 still needs further development [164]. Recent studies have shown that patients receiving tocilizumab have a better prognosis in early COVID-19 infection, and that administration of tocilizumab after the onset of acute respiratory distress syndrome (ARDS) can reduce mortality [165,166,167]. Studies have shown that tocilizumab can improve the ventilation status of mechanically ventilated patients with COVID-19 infection and significantly prolong the number of ventilator-free days, compared with the non-tocilizumab group, the average prolongation of ventilator-free days can be up to 4.7 days [168]. Recently, Chowdhry et al., also proposed that infusion of tocilizumab in intensive care unit (ICU) patients with COVID-19 significantly improved survival compared with non-tocilizumab [169]. Notably, investigators have suggested that the combination of plasma and tocilizumab during rehabilitation is associated with higher patient survival compared with tocilizumab alone [169]. This result suggests the importance of comprehensive treatment for COVID-19 infection. In addition, tocilizumab is approved not only for chronic and acute inflammatory diseases, but also for diseases caused by autoantibodies, suggesting that tocilizumab may have therapeutic implications in other complications of COVID-19 [170]. In fact, the efficacy of tocilizumab for COVID-19 infection in rheumatoid arthritis patients has been demonstrated by numerous case reports [171,172,173]. A case report stated that a patient with Unverricht–Lundborg disease developed refractory relapsing status epilepticus during COVID-19 infection, which was eventually managed with tocilizumab [174]. In any case, tocilizumab targeting IL-6R is an effective treatment for COVID-19, but the difficulty in conducting clinical trials is that the use of tocilizumab makes it difficult to display the original inflammatory response, which hides the as the disease progresses and there are a wide variety of inflammatory factors in the body, it is difficult to completely relieve the disease with tocilizumab alone. In addition, further combination regimens are needed in the future to target various complications during COVID-19.

#### 4.3.2. Anti IL-1

Besides IL-6, IL-1, as a pleiotropic cytokine, plays an important role in various biological processes, especially in immunity, inflammation, and hematopoiesis [175]. Studies have shown that IL-1 levels are elevated during COVID-19 and lead to severe organ damage [176]. In addition, genome-wide association studies have revealed that the most important loci associated with COVID-19 severity were located in TMEM189-UBE2V1, which is involved in the IL-1 signaling pathway [177]. The above data suggest the involvement of IL-1 in tissue damage and inflammatory response during COVID-19 infection. Therefore, similar to tocilizumab, the use of IL-1 receptor antagonists (Anakinra) can also inhibit the exacerbation and mortality of COVID-19 [178,179]. In a phase 3 clinical trial, the use of anakinra resulted in lower 28-day mortality and shorter hospital stays in COVID-19 patients [180]. In addition, for severe COVID-19 patients, cohort studies found that anakinra could effectively reduce the clinical symptoms of excessive inflammation in critically ill COVID-19 patients [181,182,183]. In addition, in patients with severe respiratory failure, early injection of anakinra reduced the degree of respiratory failure and restored the pro-inflammatory/anti-inflammatory balance [184]. This has high clinical significance for COVID-19 patients with acute respiratory failure. Although a large number of current research results show that anakinra has a high therapeutic value during COVID-19, its safety remains to be explored. Studies have shown that after subcutaneous injection of anakinra 100 mg per day, 13% of patients have elevated liver transaminases, suggesting the potential risk of liver damage with anakinra [182]. In addition, similar to tocilizumab, anakinra may also have certain therapeutic significance for COVID-19 patients with rheumatoid arthritis, but studies are needed to confirm [185].

#### 4.3.3. Anti TNF-α

TNF-α is mainly produced by activated macrophages, T lymphocytes, and natural killer (NK) cells. It is rapidly released by mast cells only after degranulation, a process that requires secretion of IgE antibodies that get embedded in the cell membrane via the Fc receptor and then bind the offending allergen at the same time. TNF-α is a typical potent pro-inflammatory cytokine and plays an important role in the activation, differentiation, growth and death of immune cells. Therefore, blocking TNF-α has therapeutic significance in autoimmune diseases or infectious diseases [186]. Some studies have pointed out that TNF-α can combine with other cytokines to play a role in tissue damage and excessive inflammation during COVID-19 [187]. Therefore, targeting TNF-α is also a potential pharmacological treatment for COVID-19. However, a randomized controlled trial showed that the use of a TNF-α inhibitor (adalimumab) did not show efficacy in severe COVID-19 patients [188]. Nevertheless, in addition to the specific targeted drug such as adalimumab, there are other drugs that may also act through suppressing TNF-α. At present, a large number of studies have confirmed the therapeutic effect of N-acetylcysteine in COVID-19, but the mechanism remains to be explored [189,190]. Recent data suggest that N-acetylcysteine may alleviate patient symptoms by reducing cytokine release, including TNF-α, during COVID-19 [191]. In addition, tramadol, as a strong analgesic, has also been reported to alleviate COVID-19 symptoms by reducing cytokine storm including TNF-α [192]. According to the current results, targeting TNF-α in the treatment of COVID-19 has a certain effect, but the current clinical evidence for adalimumab is still insufficient, and a large number of in vitro and in vivo experiments are still needed to verify whether adalimumab is truly effective against COVID-19.

At present, the treatment of COVID-19 with cytokines has been proposed, but according to the results of existing clinical trials and the huge number of inflammatory factors during infection, it may be necessary to combine multiple cytokine inhibitors to play a therapeutic role in the future. Evidence for combination therapy with multi-targeted cytokines is still insufficient.

### 4.4. M^Pro^

The virus begins to replicate after entering the host cell. When the replication reaches a certain amount, the progeny virus is released from the cell to achieve the purpose of infection. Therefore, the key enzymes that inhibit virus replication can also achieve the purpose of inhibiting SARS-CoV-2 infection. The major protease M^pro^ is one of the key enzymes in the viral replication process [193]. M^pro^ is mainly responsible for the hydrolytic processing of non-structural proteins (NSP), especially NSP4-NSP16, during viral replication [194]. Inhibitors against M^pro^ are currently under development. Nirmatrelvir, the first orally bioavailable inhibitor of SARS-CoV-2 M^pro^ [195], was shown in in vitro studies to inhibit SARS-CoV-2 virus replication in differentiated normal human bronchial epithelial cells. The bronchial epithelial cells were treated with different concentrations of nimarivir for three days, showing no significant cytotoxicity [193]. Moreover, twice-daily doses of 300 and 1000 mg/kg nirmatrelvir were effective in reducing the viral load in the lungs of mice 10 days after SARS-CoV-2 infection [193]. These exciting data have led to its emergence as a promising candidate for further clinical trials. One clinical trial showed that a five-day regimen of nirmatrelvir significantly reduced viral replication [196]. Additional clinical trials have shown that treatment of symptomatic COVID-19 patients with nirmatrelvir plus ritonavir results in an 89% lower risk of progression to severe COVID-19 than placebo, with no apparent safety concerns [193].

**Figure 2:**
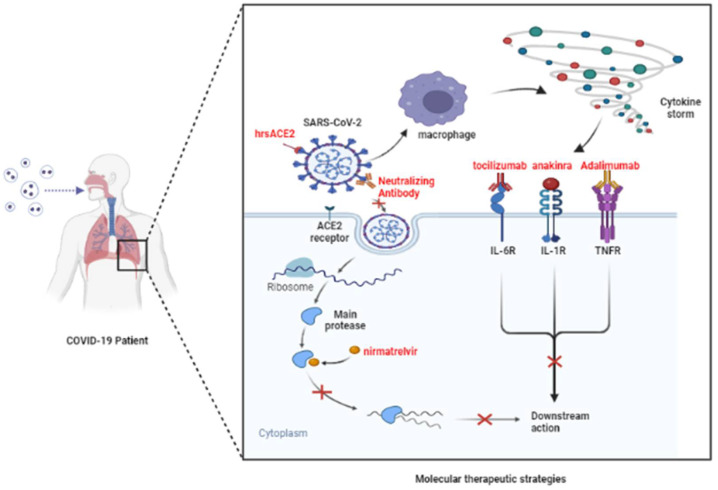
**Potential molecular therapeutic mechanisms.** SARS-CoV-2 can enter cells and replicate proteins by binding to ACE2 receptors. In addition, the virus in the alveoli also stimulates the immune system to produce a cytokine storm, which binds to receptors. It does this by blocking the viral S protein and the ACE2 receptor, or by inhibiting the activity of the main protease, inhibiting cytokine receptors and other strategies, and then producing an antiviral effect. (*Adapted from “COVID-19 Drug Mechanism of Action (Layout)”, by BioRender.com (2022). Retrieved from https://app.biorender.com/biorender-templates* (accessed on 28 July 2022).

It is also noteworthy that nirmatrelvir has been shown to be effective against different variants of SARS-CoV-2. Some studies have pointed out that nirmatrelvir inhibited the main variants including Beta and Lambda variants to a similar extent as the WT variant, with <50% inhibitory activity at 5 nM, >50% inhibition at 20 nM, and 100 nM completely inhibited SARS-CoV-2 enzyme activity of the Beta and Lambda strain [197]. In addition, nimarprevir (250 mg/kg, twice daily) in mice completely protected mice from nasal infection with Delta variants [198]. However, the current evidence for nirmatrelvir in the treatment of Omicron strains of infection is still limited (Figure 2).

### 4.5. TMPRSS2

The transmembrane protease serine 2 (TMPRSS2) is considered to be one of the major host factors responsible for the virulence of SARS-CoV-2 and the pathogenesis of COVID-19. When the S protein of SARS-CoV-2 binds to ACE2 on the surface of the host cell membrane, TMPRSS2 cleaves ACE2 and the S protein, and then the entire host–pathogen interaction begins [199]. Therefore, inhibition of host cell TMPRSS2 could serve as a potential anti-SARS-CoV-2 intervention. In addition, a case-control study of 213 SARS-CoV-2-positive individuals found that nasopharyngeal TMPRSS2 was associated with respiratory distress between COVID-19 [200]. This further supports the potential therapeutic significance of TMPRSS2 in COVID-19. In fact, in vitro experiments show that camostat, as a TMPRSS2 inhibitor, can effectively inhibit virus-cell membrane fusion. In VeroE6 cells, treatment with camostat effectively reduced the cell fusion efficiency of the Delta variant [201]. In addition, the metabolite 4-(4-guanidinobenzoyloxy) phenylacetic acid (GBPA) of camostat mesylate was also shown to have similar antiviral activity [202]. Clinical trial results showed that the camostat group resolved COVID-19 symptoms faster and improved loss of taste and smell compared to the placebo group [203]. Moreover, the combination of oral favipiravir, camostat, and ciclesonide can reduce the length of hospital stay in patients with moderate COVID-19 without safety concerns [204]. Although most of the combination drugs have high efficacy, the comparison of the efficacy of combination drugs and camostat alone is still relatively limited. Interestingly, camostat was not effective against SARS-CoV-2 infection in an earlier study [205]. The reason for this difference is currently unknown, and the efficacy of camostat alone needs to be further explored in the future. At the pharmacological level, nafamostat acts as a more specific TMPRSS2 inhibitor that inhibits virus–cell membrane fusion at lower concentrations than camostat mesylate [206]. Animal experiments have also shown that nafamostat reduces SARS-CoV-2 lung infection in a mouse model of COVID-19 [207]. At present, potential drugs against TMPRSS2 include camostat and nafamostat, but clinical studies are still limited and the efficacy and safety of these two drugs will continue to be evaluated in the clinic in the future, while a lot of basic research is needed to understand their mechanisms of action.

## 5. Conclusions

The COVID-19 outbreak caused by SARS-CoV-2 has been ongoing for almost three years. As the virus has continued to spread and evolve, more and more variants have appeared. Although a variety of vaccines are currently available to prevent COVID-19 caused by different strains of infection, a unified and effective treatment regimen has been lacking. Here, we reviewed literature on current mainstream variants, the widely used vaccines, and different therapeutic targets (viral S protein, ACE2, cytokines, major proteases, and TMPRSS2) in an attempt to provide better preventive and treatment strategies for different SARS-CoV-2 variants.

## Figures and Tables

**Table 1:** Information of main SARS-CoV-2 variants.

Variants	Strain Name	First Reported Place	First Reported Time	Main S Protein Mutation
Alpha	B.1.1.7	United Kingdom	December 2020	ΔH69, ΔV70, Δ144, N501Y, A570D, D614G, P681H, T716I, S982A, D1118H
Beta	B.1.351	South Africa	October 2020	D80A, D215G, Δ241, Δ242, Δ243, V367F, P384L, R408I, K417N, E484K, N501Y, D614G, A701V
Gamma	P.1	Japan/Brazil	January 2020	L18F, T20N, P26S, D138Y, R190S, K417T, E484K, N501Y, D614G, H655Y, T1027I, V1176F
Delta	B.1.617.2	India	December 2020	T19R, T95I, G142D, R158G, K417N, L452R, T478K, D614G, P681R, D950N
Omicron	B.1.1.529	South Africa	November 2021	A67V, ΔH69, ΔV70, T95I, G142D, ΔV143, ΔY144, ΔY145, ΔN211, L212I, ins214EPE, G339D, K417N, N440K, G446S, S477N, T478K, E484A, Q493R, G496S, Q498R, N501Y, Y505H, T547K, D614G, N679K, P681H, N764K, D796Y, Q954H, N969K, L981F, and N856K
Lambda	C.37/B.1.1.1	Peru	August 2020	G75V, T76I, Δ246–252 (1246–252), L452Q, F490S, D614G and T859N
Mu	B.1.621	Colombia	January 2021	Y144T, Y145S, R346K, E484K, N501Y and P681H
Kappa	B.1.617.1	India	December 2021	L452R, T478K, E484Q, D614G, and P681R

Δ indicates deletion.

**Table 2:** Basic information of current vaccines.

Vaccine	Platform	Antigen	Efficacy of Infection	Immune Type	Sponsor Location
Pfizer/BioNTech mRNA vaccine	mRNA	Full-length S protein	95% in WT infection [77]; 89.5–93.7% (Alpha) [121]; 75–100% (Beta) [77]; 52.4–88% (Delta) [88]; 22.5% (Omicron) [122]	Humoral immunity	Germany
Moderna mRNA-1273 vaccine	mRNA	Segments of SARS-CoV-2 hereditary material/ Stabilized Spike	94.1% in WT infection [85]; 89% (Alpha); 85% (Gamma) [87]; 96.4% (Beta) [86]; 50.6% (Delta) [88]	Humoral and cellular immunity	USA
AZD1222 vaccine	Viral vector	Whole-length S protein	62.1–79% in WT infection [93,94]; 74.5 (Alpha); 67% (Delta) [96]; 10.4% (Beta) [95]	Cellular immunity	UK
CoronaVac vaccine	Inactivated virus	Inactivated whole SARS-CoV-2 virus	83.5% in WT infection in patients aged 18–59 [106]; 75–88.1% (Alpha), 64.2–70% (Beta), 88.1% (Gamma), 48.33–78.6% (Delta) [107]	Humoral immunity	China
DNA vaccine	DNA	Plasmid DNA carrying S gene of SARS-CoV-2	66.6% of ZyCoV-D vaccine in WT infection [115]	Humoral and cellular immunity	USA/India
NVX CoV-2373 vaccine	Protein subunit	SARS-CoV-2 S protein	89.7% in WT infection [116]; 86.3% (Alpha) [116]; 43% (Gamma) [87]	Humoral immunity	USA

## Data Availability

Not applicable.
